# Integrative bioinformatics and machine learning identify iron metabolism genes MAP4, GPT, and HIRIP3 as diagnostic biomarkers and therapeutic targets in Alzheimer’s disease

**DOI:** 10.3389/fncel.2025.1610682

**Published:** 2025-06-06

**Authors:** Xiaoqiong An, Xiangguang Zeng, Zhenzhen Yi, Manni Cao, Yijia Wang, Wenfeng Yu, Zhenkui Ren

**Affiliations:** ^1^Department of Laboratory Medicine, The Second People’s Hospital of Guizhou Province, Guiyang, China; ^2^Key Laboratory of Molecular Biology, Guizhou Medical University, Guiyang, China; ^3^Department of Laboratory Medicine, Qianxinan People’s Hospital, Xingyi, China; ^4^Center for Tissue Engineering and Stem Cell Research, Guizhou Medical University, Guiyang, China; ^5^Key Laboratory of Human Brain Bank for Functions and Diseases of Department of Education of Guizhou Province, Guizhou Medical University, Guiyang, China

**Keywords:** Alzheimer’s disease, iron metabolism, GPT, MAP4, HIRIP3, machine learning

## Abstract

**Background:**

Alzheimer’s disease (AD) is a progressive neurodegenerative disorder characterized by cognitive decline, memory impairment, and the accumulation of pathological markers such as amyloid-beta plaques and neurofibrillary tangles. Recent evidence suggests a role for dysregulated iron metabolism in the pathogenesis of AD, although the precise molecular mechanisms remain largely undefined.

**Materials and methods:**

To address the role of iron metabolism in AD, we utilized an integrative bioinformatics approach that combines weighted gene co-expression network analysis (WGCNA) with machine learning techniques, including LASSO regression and Generalized Linear Models (GLM), to identify hub genes associated with AD. We used transcriptomic data derived from postmortem prefrontal cortex samples (GSE132903, comprising 97 AD cases and 98 controls). To assess changes in the immune microenvironment, single-sample gene set enrichment analysis (ssGSEA) was employed. Furthermore, pathway enrichment analysis and gene set variation analysis (GSVA) were performed to uncover the underlying biological mechanisms driving these alterations. Protein validation was carried out in APP/PS1 transgenic mice through Western blotting.

**Results:**

Three genes related to iron metabolism—MAP4, GPT, and HIRIP3—are identified as strong biomarkers. The GLM classifier showed high diagnostic accuracy (AUC=0.879). AD samples had increased immune activity, with more M1 macrophages and neutrophils, indicating neuroinflammation. MAP4 and GPT were linked to Notch signaling and metabolic issues. In APP/PS1 mice, MAP4 decreased, while GPT and HIRIP3 increased.

**Discussion:**

This analysis highlights these genes as diagnostic biomarkers and therapeutic targets, connecting iron balance, neuroinflammation, and metabolic problems in AD. The immune profile suggests potential for immunomodulatory treatments, enhancing understanding of AD and aiding precision diagnostics and therapies.

## Introduction

AD, a chronic neurodegenerative condition characterized by progressive synaptic failure and cognitive decline, represents a global public health crisis with profound socioeconomic implications (Zheng and Wang, 2025; Khemka et al., 2023). While amyloid-β plaques and neurofibrillary tangles remain diagnostic hallmarks, contemporary research reveals intricate pathophysiological networks involving neuroimmune crosstalk, mitochondrial dysfunction, and epigenetic modifications (Liu et al., 2023; Um et al., 2024; Migliore and Coppedè, 2022). Emerging evidence further implicates dysregulated iron homeostasis and lipid peroxidation cascades in AD progression, suggesting novel therapeutic targets (Lane et al., 2021; Lou et al., 2021). Despite these advances, critical gaps persist in understanding molecular dynamics and developing clinically reasonable biomarkers, highlighting the imperative for integrated multi-omics approaches to decode AD’s biological complexity.

Current diagnostic methods for AD mainly depend on clinical assessments and imaging, which often miss early detection (Khan et al., 2020; Graff-Radford et al., 2021). Treatment options are limited, creating a need for biomarkers to aid early diagnosis and targeted interventions. Advances in bioinformatics and molecular biology have identified potential AD biomarkers, but integrating these into clinical practice is challenging (Bai et al., 2021; Bai et al., 2020). Recent research highlights iron metabolism’s role in AD, linking iron imbalance to neuronal degeneration and identifying key genes involved (Wu et al., 2023; Wang et al., 2023). These findings offer potential new therapeutic targets, but further studies are needed to confirm their clinical significance.

This study introduces a comprehensive bioinformatics framework that combines ensemble machine learning techniques with weighted gene co-expression network analysis (WGCNA) to explore molecular networks associated with AD and uncover critical regulatory pathways (Feng et al., 2025; Lian et al., 2023). This computational paradigm enables systematic mining of high-dimensional multi-omics datasets (Wu et al., 2024). The ultimate translational objective is to construct a clinically interpretable, individualized risk stratification nomogram based on mechanistically relevant biomarkers, facilitating precision early diagnosis and mechanism-guided therapeutic development for AD.

Moreover, the investigation of immune responses in AD has gained traction (Chen and Holtzman, 2022), as recent research indicates an altered immune landscape in patients, characterized by the activation of specific immune cell types (Gao et al., 2023; Koronyo et al., 2023). Understanding these immune interactions is vital for developing immunotherapeutic strategies aimed at mitigating neurodegeneration.

This research primarily aims to elucidate key genes related to iron metabolism and their interactions within the immune microenvironment. By leveraging bioinformatics approaches, we aspire to provide an in-depth characterization of the molecular changes occurring in AD, which will help facilitate the identification of novel biomarkers and potential therapeutic targets. The ultimate objective of this study is to contribute to the development of more precise diagnostic tools and targeted treatment strategies for AD, addressing the critical need for improved management of this debilitating condition.

## Methods

### Data acquisition

To investigate molecular alterations underlying neurodegenerative pathology, the current study employed a well-characterized cohort of post-mortem transcriptomic profiles (97 AD vs. 98 controls) obtained from the Gene Expression Omnibus (GEO) repository under accession number GSE132903. This publicly archived dataset, hosted by the National Center for Biotechnology Information (NCBI).

### Weighted gene co-expression network analysis

Weighted gene co-expression network analysis (WGCNA) was employed to identify gene modules linked to AD using gene expression data. The co-expression network for the GSE132903 cohort was established following the scale-free topology criterion. We first calculated the Pearson correlation coefficient to generate a similarity matrix, which was subsequently transformed into an adjacency matrix by applying a soft threshold of 19. This adjacency matrix was further transformed into a topological overlap matrix (TOM) to facilitate average-linkage hierarchical clustering, enabling the delineation of gene modules with a minimum of 10 genes. By merging similar gene modules, we identified a total of nine distinct co-expressed gene modules, designating the light cyan module as the core module. Additionally, the “VennDiagram” package was utilized to visualize the overlap between iron metabolism associated genes and genes within the light cyan module.

### Construction machine learning algorithm

To identify key genes significantly linked to the development of AD, we implemented a systematic computational framework that integrates four distinct machine learning algorithms: generalized linear modeling (GLM), support vector machine (SVM), random forest (RF), and extreme gradient boosting (XGBoost). Model optimization was achieved through rigorous hyperparameter tuning and cross-validation, with predictive performance quantified via ROC curve analysis and residual distribution profiling. Through comparative performance evaluation, three high-confidence biomarker candidates (MAP4, GPT, and HIRIP3) emerged as hub genes based on multi-criteria feature selection incorporating permutation importance and SHAP value rankings. The discriminatory capacity of these biomarkers was subsequently validated through ROC-AUC quantification.

### Establishment of a nomogram model for AD diagnosis

Based on the three key genes identified through machine learning, we established a nomogram model using the “rms” R package to evaluate the risk of AD. This diagnostic nomogram was designed to evaluate AD occurrence and assess risk in patient groups. Each predictor in the nomogram contributes a specific score, and the “total score” is calculated as the sum of these individual scores. To validate the reliability of our predictions, we applied a calibration curve to measure the consistency between predicted values and actual observations. Additionally, we generated a ROC curve and computed the AUC value using the “pROC” R package to evaluate the model’s predictive accuracy. Furthermore, we performed a decision curve analysis (DCA) to assess the clinical benefit of model-based decision-making for patients.

### Immune microenvironment analysis

To comprehensively explore the differences in the immune microenvironment between AD and normal groups, we utilized single-sample gene set enrichment analysis (ssGSEA) through the “GSVA” package. This approach quantified the infiltration levels of 28 immune cell types within the AD samples, providing insights into the immune landscape associated with AD pathogenesis. The expression distribution of these immune cells was visually depicted through box diagram for both AD and control groups. Furthermore, we performed Pearson correlation analysis to investigate the relationship between the expression levels of three key hub genes (MAP4, GPT, and HIRIP3) and immune infiltration levels. To evaluate the influence of these hub genes on immune cell infiltration, we also employed boxplots to compare infiltration levels between groups with high and low expression of these genes.

### Single-gene gene set enrichment analysis of key hub genes

Gene set enrichment analysis (GSEA) is a computational approach employed to assess the functional annotation of gene sets and ascertain their significance within specific biological contexts. Following the identification of three key hub genes—MAP4, GPT, and HIRIP3—single-gene GSEA was conducted for each gene across both groups. This analysis sought to investigate the biological functions, signaling pathways, and regulatory roles of the hub genes, thereby elucidating their molecular mechanisms and functional significance. Pathways with a *p*-value less than 0.05 were deemed statistically significant. The three most notable up-regulated and down-regulated pathways were subsequently highlighted.

### Gene set variation analysis

To analyze the differential enrichment of KEGG pathways and Gene Ontology (GO) terms between the AD and normal groups, we applied gene set variation analysis (GSVA) in this study. The GSVA R package was utilized in conjunction with curated gene sets, specifically GO terms, sourced from the Molecular Signatures Database (MSigDB). Annotation files for KEGG pathways and GO terms were acquired from the official MSigDB repository. Statistical significance for enriched terms was determined using a threshold of *p* < 0.05.

### Animals

Male C57BL/6J (wild-type, 6-month-old) and APP/PS1 double-transgenic murine models were sourced commercially from SLAC Laboratory Animal Center (Shanghai, China). This experimental protocol received formal ethical approval from the Institutional Animal Care Committee at Guizhou Provincial Second People’s Hospital (Ethics Approval Code: 202102). All subjects were housed in standardized conditions (21 ± 2°C ambient temperature; 45–55% relative humidity; 12/12 h photoperiod) with ad libitum access to autoclaved feed and reverse-osmosis water. Post-euthanasia via carbon dioxide asphyxiation, whole-brain specimens were rapidly harvested through craniotomy. Hippocampal tissues were carefully microdissected under RNase-free conditions and then flash-frozen in liquid nitrogen at −80°C to preserve them for subsequent analyses.

### Western blot

SDS-PAGE was used to separate proteins by loading 20 μg of each sample onto a 12% polyacrylamide gel. Following electrophoresis, the proteins were transferred onto a 0.2 μm nitrocellulose membrane. After blocking with 5% non-fat milk for 2 h at room temperature, the membrane was incubated overnight at 4°C with primary antibodies against MAP4 (1:2,000; Proteintech), GPT (1:2,000; Proteintech), and HIRIP3 (1:1,000; Proteintech). Following TBST washes, the membrane was incubated with a secondary antibody for 2 h at room temperature. Protein detection was performed using ECL chemiluminescent reagents.

## Results

### Identification of co-expression gene modules in AD

**Figure 1:**
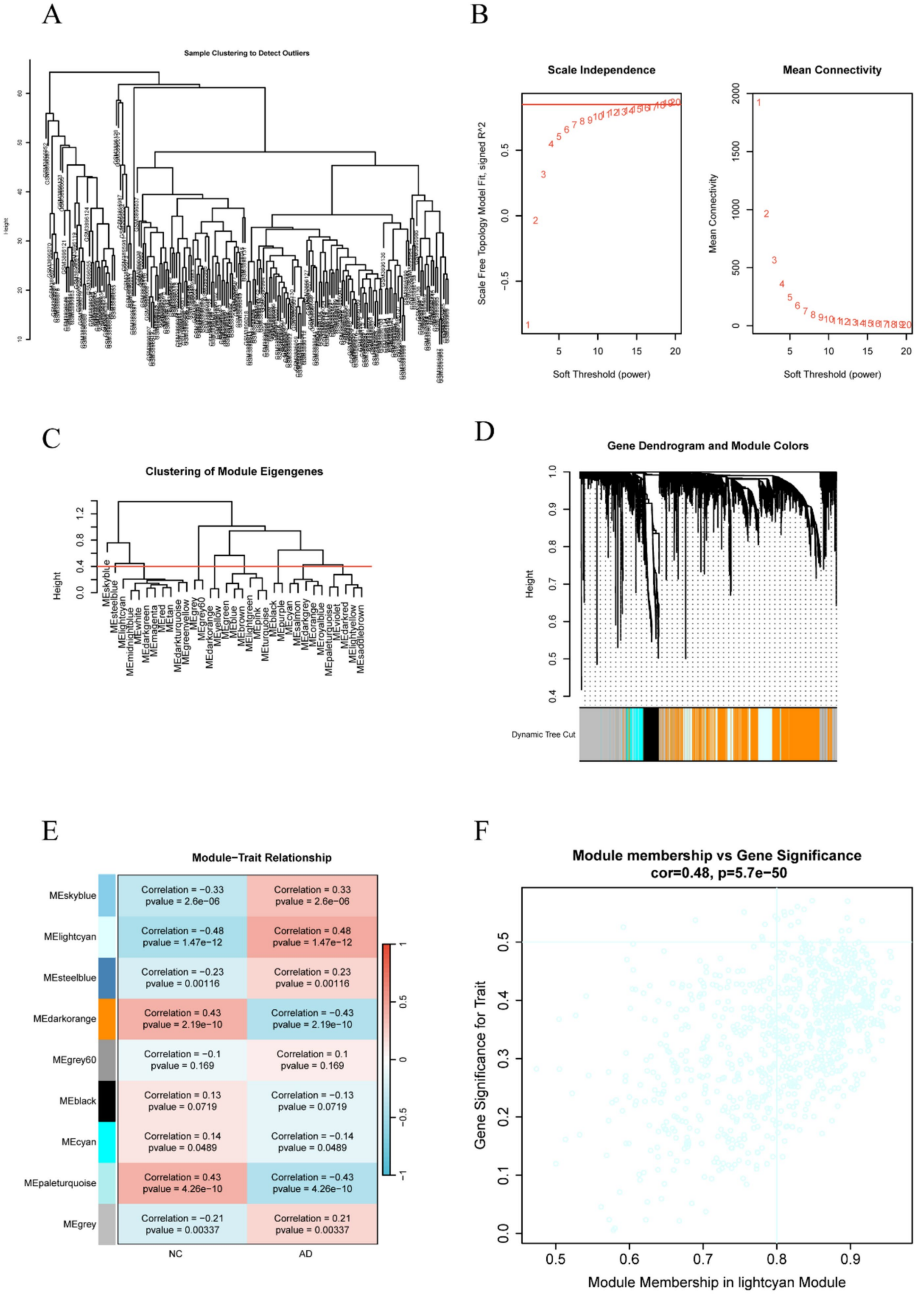
Construction of a co-expression network and identification of model-related genes associated with AD using WGCNA. **(A)** Sample clustering to detect outliers. **(B)** Exploration of network topology in the context of diverse soft thresholds (β). **(C)** Clustering of module eigengenes. **(D)** Gene dendrogram and module colors. **(E)** Module–trait relationships: each cell shows the related correlation and *p*-value. **(F)** A scatterplot of module membership versus gene significance membership in the light cyan module.

Firstly, we performed sample clustering to construct a sample tree, which is depicted in Figure 1A. This step was crucial for identifying and removing outlier samples that could potentially skew the results. Following the removal of the outliers, we proceeded to determine the optimal soft threshold for constructing a scale-free network. The soft threshold is a critical parameter in WGCNA that helps define the adjacency matrix and, consequently, the network topology. We tested various soft thresholds and found that a scale independence of > 0.85 indicated that a soft threshold power (*β*) of 19 was optimal for our dataset, as shown in Figure 1B. Through hierarchical clustering and dynamic tree cut, we identified 10 distinct gene modules, which are illustrated in Figures 1C,D. Among these, the light cyan module (*r* = 0.48; *p* = 1.47 × 10^−12^), the dark orange module (*r* = 0.43; *p* = 2.19 × 10^−10^), and the pale turquoise module (*r* = 0.43; *p* = 4.26 × 10^−10^) exhibited the highest positive associations with AD, as depicted in Figure 1E. These modules contain genes that may play crucial biological roles in the disease’s signature. Notably, the light cyan module showed the strongest correlation with AD, leading us to focus on it for further analysis. The scatterplot revealed a high correlation between module membership (MM) and gene significance (GS) in the light cyan module (Figure 1F).

### Three genes were screened as AD key genes

**Figure 2:**
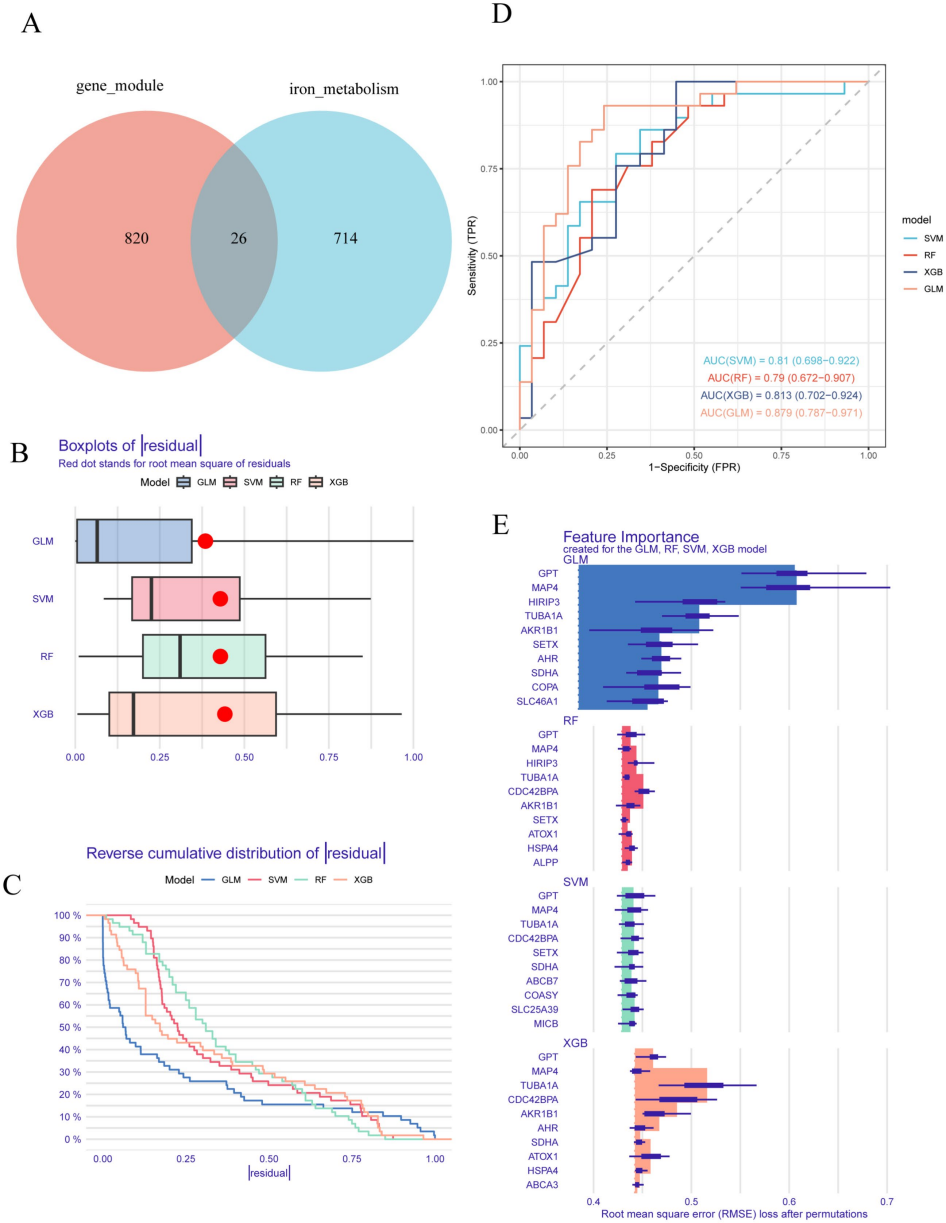
Establishment and evaluation of RF, SVM, GLM, and XGB machine models. **(A)** A Venn diagram of light cyan module genes versus “iron metabolism” associated genes. **(B)** Boxplots illustrated the residuals of each machine learning model, with the red dot marking the root mean square error (RMSE). **(C)** Cumulative residual distribution of each machine learning model. **(D)** The ROC curves predict the accuracy of each learning machine model. **(E)** The important features in SVM, RF, GLM, and XGB machine models.

To discover novel biomarkers associated with differential expression in AD, we searched the GeneCards database using the term “iron metabolism,” retrieving a total of 740 genes. A Venn diagram analysis was then performed to evaluate the overlap between these “iron metabolism-related genes” and those in the light cyan module, leading to the identification of 26 core genes (Figure 2A). Subsequently, we employed machine learning algorithms to develop four diagnostic models, including the generalized linear model (GLM), random forest (RF), XGBoost (XGB), and support vector machine (SVM), with the objective of identifying hub genes possessing diagnostic significance for AD. As illustrated in Figures 2B,C, the GLM model demonstrates a smaller residual distribution in comparison to the SVM, RF, and XGB models. Furthermore, the performance of the four models was assessed using receiver operating characteristic (ROC) curves. Among these models, the generalized linear model (GLM) demonstrated the highest area under the curve (AUC) value of 0.879, indicating superior predictive accuracy compared to other machine learning algorithms (Figure 2D). Subsequently, the top 10 characteristic genes for each model were ranked according to their root mean square error (RMSE) (refer to Figure 2E). Ultimately, based on the previously discussed results, the GLM was identified as the most effective method for distinguishing patients with varying patterns. Consequently, the top three critical genes—MAP4, GPT, and HIRIP3—were selected for inclusion in the subsequent diagnostic prediction model.

### Construction of a nomogram model

**Figure 3:**
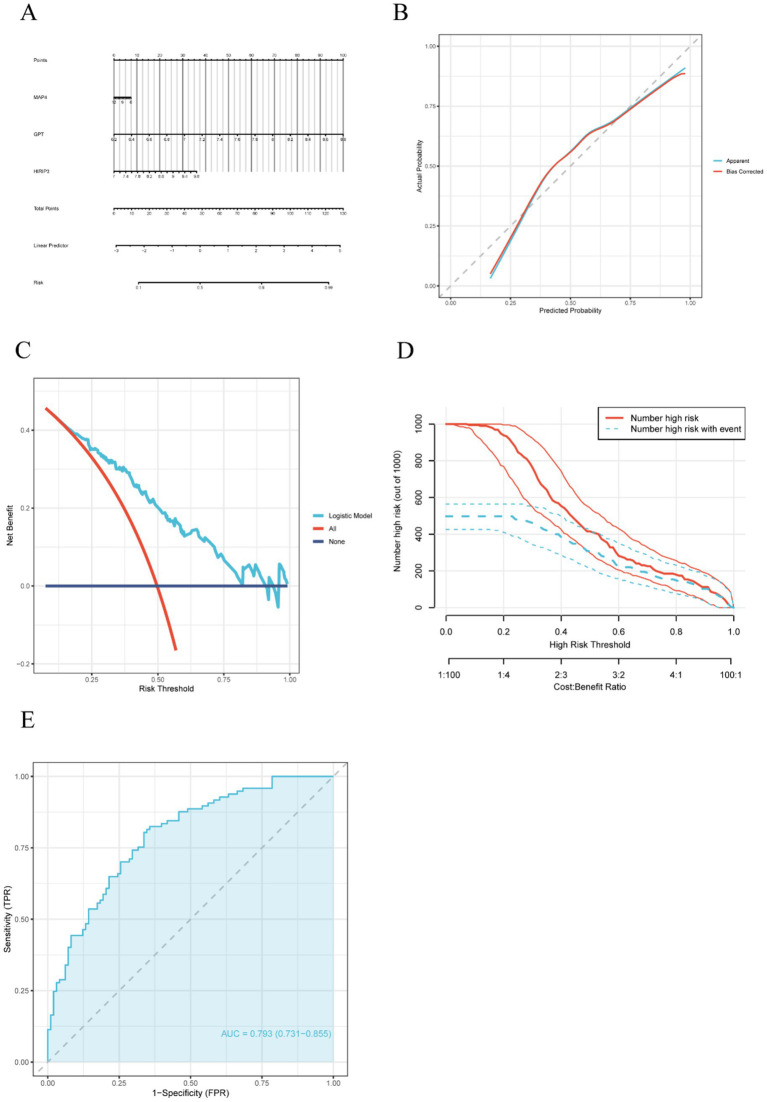
Construction of a nomogram model for AD diagnosis. **(A)** The nomogram of diagnostic biomarkers predicts the occurrence of AD based on three model-related genes. **(B)** A calibration curve was applied to assess the predictive accuracy of the nomogram model. **(C)** The DCA curve assesses the clinical utility of the nomogram model. **(D)** The clinical impact curve illustrates the effect of the predictive model on clinical outcomes. **(E)** The ROC curve demonstrated the predictive accuracy of the nomogram model in the training set.

To evaluate the risk associated with AD and further validate the predictive efficacy of the three hub genes (MAP4, GPT, and HIRIP3), we employed the “rms” package to construct a nomogram incorporating these genes (Figure 3A). The calibration curve illustrated the model’s strong calibration, indicating a high degree of accuracy in forecasting the risk of AD (Figure 3B). Additionally, the DCA revealed that within a threshold probability spectrum ranging from 0.2 to 0.8, patients who employed this model experienced greater advantages compared to those who did not receive any intervention or those who underwent complete intervention (Figure 3C). The clinical impact of the nomogram was further evaluated through DCA. In the high-risk threshold range of 0.4 to 1, the curve representing “high risk number” closely paralleled the curve for “high risk with event number” (Figure 3D). This substantial alignment implies that the nomogram possesses considerable predictive capability. Moreover, we also created a receiver operating characteristic (ROC) curve and determined the area under the curve (AUC) using the GSE132903 dataset to evaluate the model’s predictive performance. An AUC value exceeding 0.7 indicates that the model possesses strong discriminative ability and demonstrates excellent accuracy in predicting AD (Figure 3E).

### Immune microenvironment differences between ad and normal groups

**Figure 4:**
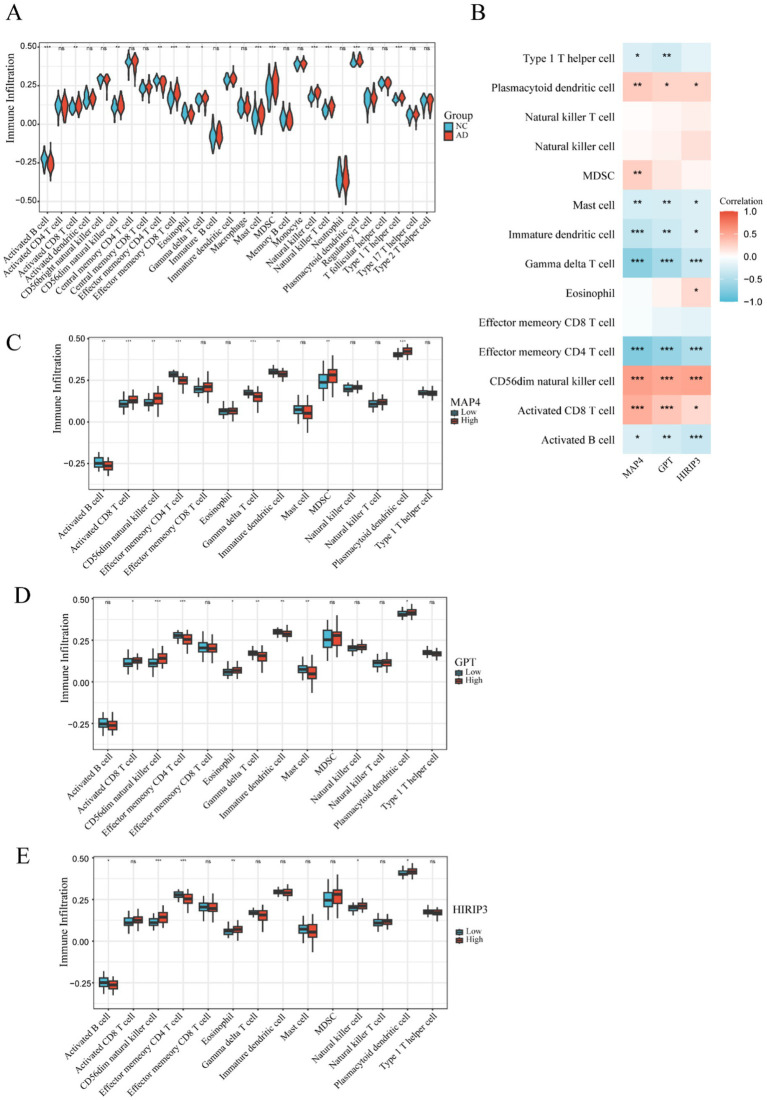
The immune microenvironment within the AD and normal groups. **(A)** The violin diagram presents the abundance of immune cells in the AD and normal groups. **(B)** The correlation of three hub genes (MAP4, GPT, and HIRIP3) with immune infiltration was assessed via Pearson correlation analysis. **(C–E)** Boxplots were used to visualize the infiltration of immune cells in groups with high and low expression of the three key hub genes (MAP4, GPT, and HIRIP3). Statistical significance was determined as ^*^p < 0.05, ^**^p < 0.01, and ^***^p < 0.001.

To elucidate the differences in the immune microenvironment, we utilized single-sample gene set enrichment analysis (ssGSEA) to examine immune infiltration in both AD and normal groups. The findings indicated that, compared to the normal group, the AD group exhibited a higher enrichment of several immune cell types, including activated B cells, activated CD8 T cells, CD56^dim^ natural killer cells, effector memory CD8 T cells, gamma delta T cells, immature dendritic cells, MDSCs, natural killer cells, natural killer T cells, type 1 T helper cells, and plasmacytoid dendritic cells (Figure 4A). Conversely, the AD group had lower enrichment of effector memory CD4 T cell, eosinophil, and mast cell (Figure 4A). In AD, effector memory CD4^+^ T cells contribute to the regulation of immune inflammation through the secretion of cytokines, facilitation of antibody production by B cells, and interactions with neurons and glial cells (Machhi et al., 2021; Kostic et al., 2025). Eosinophils release toxic proteins and cytokines that damage nerve cells and contribute to Aβ deposition (Zhang et al., 2022). Mast cells, once activated, release inflammatory mediators, interact with microglia and other cells, and regulate the neuroimmune network (Skaper et al., 2017). These results demonstrate that immune infiltration in the brain may play a role in promoting the development of AD.

In addition, a correlation heatmap illustrated the relationships between the three hub genes (MAP4, GPT, and HIRIP3) and immune cells (Figure 4B). The correlation analysis showed that CD56^dim^ natural killer cell, activated CD8 T cell and plasmacytoid dendritic cell were positively associated with MAP4, GPT, and HIRIP3, while, mast cell, immature dendritic cell, gamma delta T cell, effector memory CD4 T cell and activated B cell were negatively associated.

Furthermore, we examined the differences in immune cell infiltration levels between high-expression and low-expression groups of each hub gene to better understand how key gene expression influences immune cell infiltration patterns and to uncover relevant biological mechanisms. The results revealed significant differences in immune cell infiltration between the high and low expression groups of the MAP4 gene (Figure 4C), including activated CD8 T cells, CD56^dim^ natural killer cells, MDSCs, plasmacytoid dendritic cells, activated B cells, effector memory CD4 T cells, gamma delta T cells, and immature dendritic cells. Analysis of the GPT gene expression groups (Figure 4D) revealed significant differences in immune cell infiltration, including activated CD8 T cells, CD56^dim^ natural killer cells, effector memory CD4 T cells, eosinophils, gamma delta T cells, immature dendritic cells, mast cells, and plasmacytoid dendritic cells. Similarly, in the HIRIP3 gene expression groups (Figure 4E), significant differences were observed in the infiltration of activated B cells, CD56^dim^ natural killer cells, effector memory CD4 T cells, eosinophils, natural killer cells, and plasmacytoid dendritic cells.

These results indicate that the expression levels of the MAP4, GPT, and HIRIP3 genes are closely related to the degree of immune infiltration, suggesting that these genes may play important roles in regulating the infiltration of immune cells into specific tissues or regions, thus influencing relevant physiological or pathological processes of AD.

### Gene set enrichment analysis of hub genes

**Figure 5:**
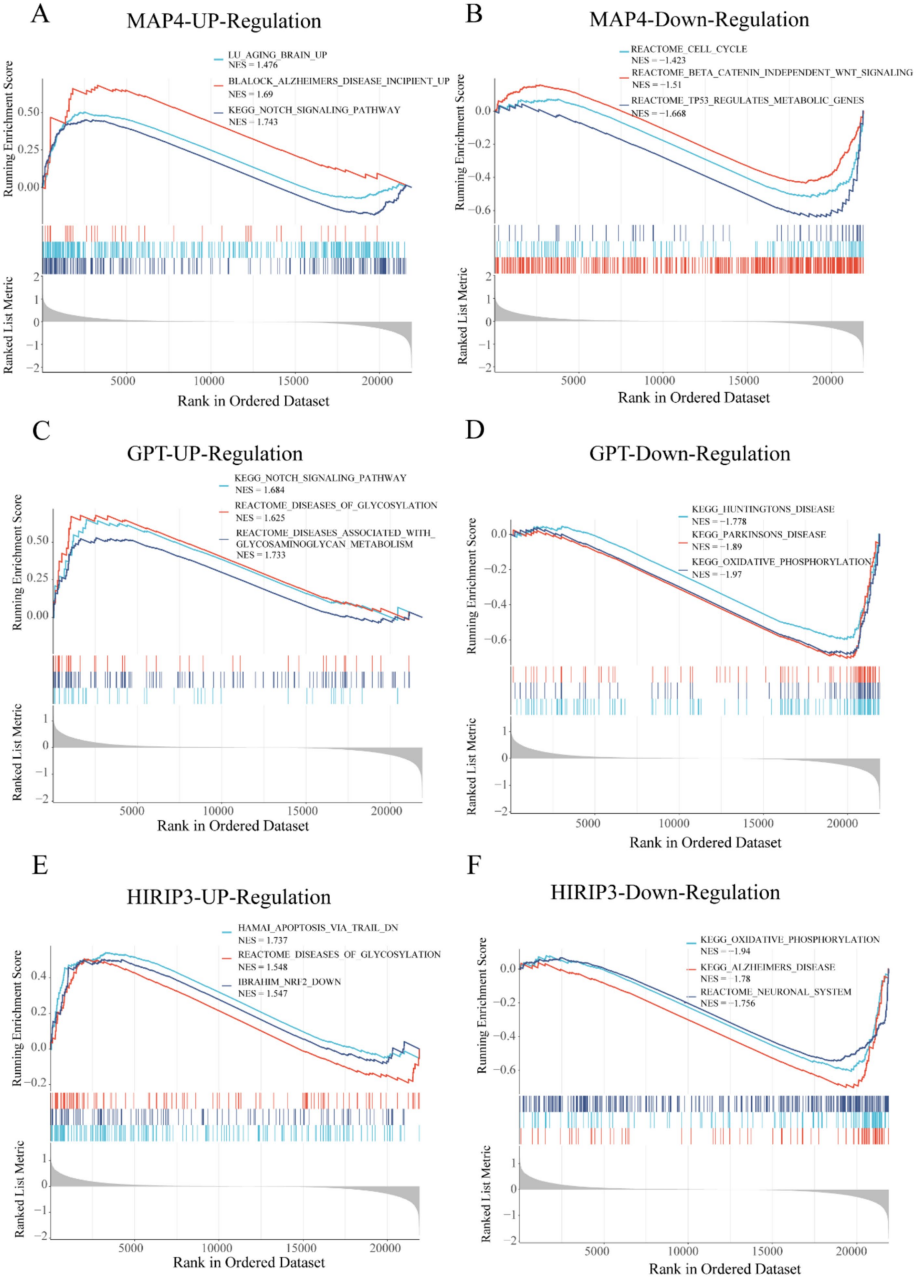
Pathway and function analysis of model-related genes. **(A,B)** The GSEA analysis identified the three pathways associated with MAP4 up-regulation and down-regulation. **(C,D)** The GSEA analysis identified the three pathways associated with GPT up-regulation and down-regulation. **(E,F)** The GSEA analysis identified the three pathways associated with HIRIP3 up-regulation and down-regulation.

To comprehensively investigate the biological functions and signaling pathways linked to the hub genes MAP4, GPT, and HIRIP3 in AD, we utilized a single-gene set enrichment analysis (GSEA) approach for pathway enrichment analysis. MAP4, GPT were positively linked with Notch_signaling_pathway. Besides, MAP4, GPT, and HIRIP3 were negatively associated with linked with some functional pathways, such as cell_cycle, Alzheimers_disease and oxidative_phosphorylation (Figures 5A–F).

### Validation of the expression levels of the three hub genes

**Figure 6:**
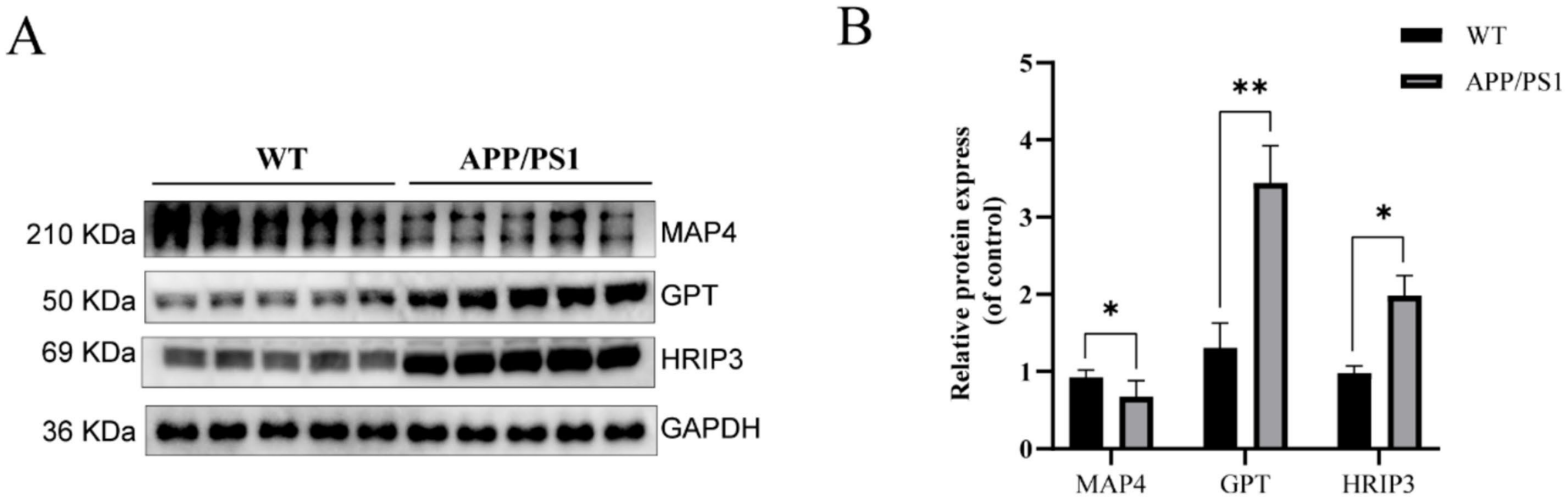
Western blot analysis confirmed the expression levels of hub genes in the APP/PS1 mouse model. **(A)** Representative immunoblots depicting the expression of MAP4, GPT, and HIRIP3 proteins in the WT and the APP/PS1 groups. **(B)** Quantification of relative protein abundance of MAP4, GPT, and HIRIP3 in the WT and APP/PS1 groups.

In order to validate the protein expression levels of the three hub genes, we conducted a western blot assay. Compared to the WT group, the protein expression level of MAP4 was downregulated, while the protein expression levels of GPT and HIRIP3 were upregulated in the APP/PS1 group (Figures 6A,B). The results were consistent with our bioinformatics analysis, reinforcing the credibility of our previous findings.

### Gene set variation analysis between control and AD groups

**Figure 7:**
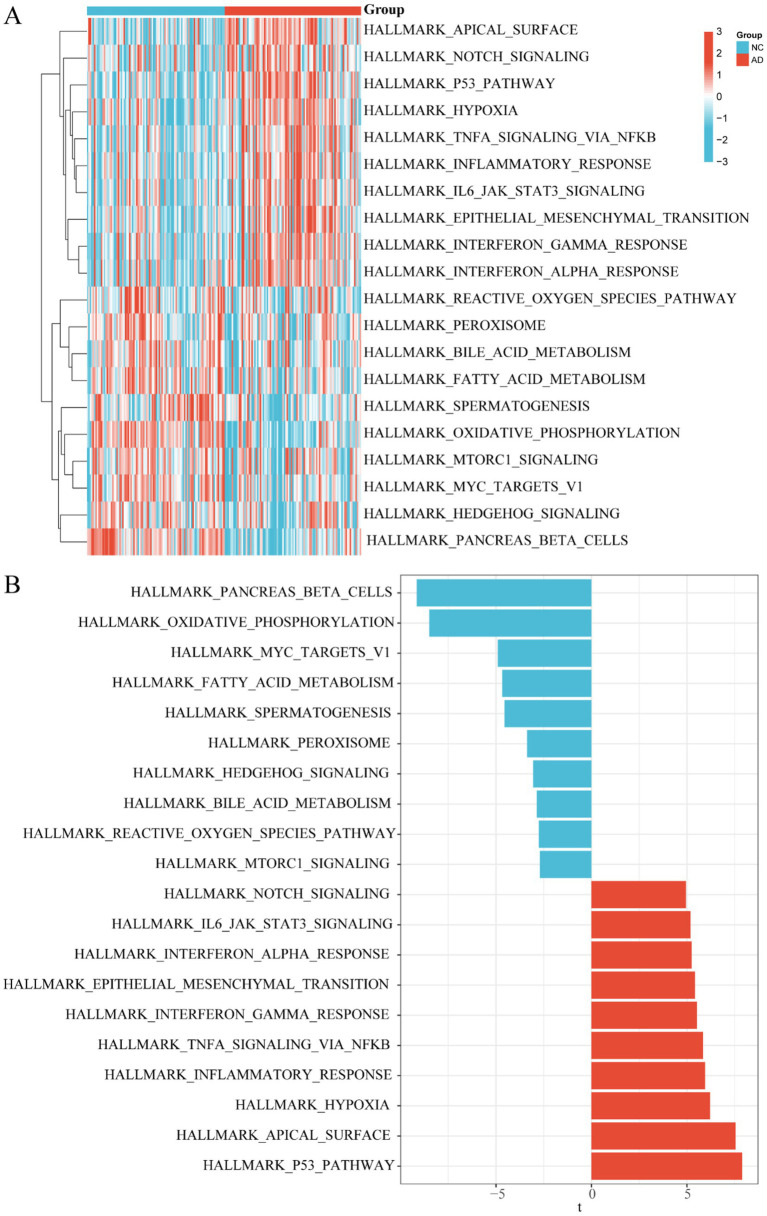
GSVA enrichment analysis in control and AD groups. **(A)** Heatmap plot between control and AD groups. **(B)** Bar chart between control and AD groups.

To thoroughly investigate the differential distribution of signal pathway enrichment within the differentially expressed “HALLMARK” gene set between the control and AD groups, we utilized the GSVA method. Through this analysis, we identified the top 10 enriched HALLMARK pathways for both up-regulation and down-regulation, which were visualized using a heatmap (*p* < 0.05) (Figure 7A). Specifically, compared to the control group, the AD group exhibited up-regulation in pathways such as notch signaling, IL6 JAK STAT3 signaling, interferon alpha and gamma responses, epithelial mesenchymal transition, TNFA signaling via NFKB, inflammatory response, hypoxia, apical surface, and P53 pathway. Conversely, down-regulated pathways in the AD group included pancreas beta cells, oxidative phosphorylation, Myc targets V1, fatty acid metabolism, spermatogenesis, peroxisome, hedgehog signaling, bile acid metabolism, reactive oxygen species pathway, and Mtorc1 signaling. For further clarity, a bar chart was also provided to illustrate the 20 differentially expressed hallmark gene sets based on GSVA score order (Figure 7B). These findings collectively underscore the complex biological dysregulations present in AD, highlighting the interplay between various pathways and processes.

## Discussion

This study aims to elucidate the molecular alterations associated with Alzheimer’s disease, focusing on key genes involved in iron metabolism and their potential role as biomarkers for diagnosis and therapy. By employing advanced bioinformatics techniques, including machine learning algorithms and weighted gene co-expression network analysis (WGCNA), we identified three hub genes—MAP4, GPT, and HIRIP3—that hold promise as diagnostic and prognostic biomarkers for AD. Genes involved in iron metabolism regulate immune cells and may influence antioxidant stress and lipid metabolism pathways (Ma et al., 2023). Especially, MAP4, a member of the microtubule-associated protein family, competes with Tau for microtubule binding, potentially influencing axonal transport in neuroinflammation (Zhang et al., 2025). These findings emphasize the importance of understanding the molecular underpinnings of AD.

The immune microenvironment analysis in AD patients showed a significant rise in activated immune cells compared to healthy controls, indicating that neuroinflammation may be key in AD’s pathogenesis. Furthermore, the analysis of the immune microenvironment in AD patients revealed a marked increase in activated immune cells compared to healthy controls (Zhang et al., 2023; Yang F. et al., 2024). This elevation in immune activity suggests that neuroinflammatory processes may play a crucial role in the pathogenesis of AD (Princiotta Cariddi et al., 2022; Uddin et al., 2022). Understanding the dynamics of immune response in the context of AD could lead to innovative therapeutic strategies, particularly those aimed at modulating immune activation to restore homeostasis in the central nervous system (Leng and Edison, 2021). The infiltration and functional activity of activated CD8 T cells and natural killer T cells may play a role in the neurodegenerative processes observed in AD (Zeng et al., 2024). Conversely, plasmacytoid dendritic cells and MDSCs have the potential to modulate the inflammatory response and neuronal damage through their influence on the local microenvironment (Salminen et al., 2018; Reizis, 2019). Furthermore, CD56^dim^ natural killer (NK) cells may contribute to AD pathogenesis via their proinflammatory responses and cytotoxic effects (Rodriguez-Mogeda et al., 2024; Ning et al., 2023).

While increased neuroinflammation and immune cell infiltration in AD have been documented, this study’s innovation lies in a correlation heatmap that reveals relationships between hub genes (MAP4, GPT, and HIRIP3) and various immune cell types. It shows positive associations with CD56^dim^ natural killer cells, activated CD8 T cells, and plasmacytoid dendritic cells, and negative associations with mast cells and others. Additionally, significant differences in immune cell infiltration levels between high and low expression groups of each hub gene suggest their critical role in modulating immune responses and related biological mechanisms.

Additionally, the pathway analysis indicates a positive correlation between MAP4 and GPT with the notch signaling pathway, while exhibiting a negative correlation with pathways associated with oxidative phosphorylation. This suggests that these genes may influence AD pathology through the modulation of critical signaling pathways. Notch pathway, which is known to affect neuronal differentiation and survival (Song et al., 2023; Giniger, 2012). Research shows that the notch pathway, activated by delta and jagged ligands, is connected to amyloid precursor protein metabolism and plaque formation, suggesting its regulatory role in Alzheimer’s disease (Yang K.-F. et al., 2024). This activation affects transcription factors such as HES and HEY, whose abnormal expression is linked to neuroinflammation and apoptosis in AD, underscoring the pathway’s complex role in the disease’s progression (Boyko et al., 2012; Leal et al., 2012). Therefore, it is plausible to hypothesize that MAP4 or GPT may play a role in the pathological mechanisms of AD via the notch signaling pathway.

The validation of findings through experimental approaches, such as western blot analysis in APP/PS1 transgenic mouse models, corroborates the potential of MAP4, GPT, and HIRIP3 as viable biomarkers and therapeutic targets for AD. MAP4 is crucial for cell migration, proliferation, and tissue remodeling by regulating microtubule dynamics, impacting both physiological and pathological states (Doki et al., 2020), it may also influence AD pathogenesis by affecting microtubule assembly and stability, thereby impacting neuron integrity and function. GPT variants disrupt neurotransmitter release, causing generalized CNS dysfunction, significant intellectual disability in patients, and MRI findings of abnormal myelin formation in subcortical white matter (Celis et al., 2015). Previous research identified HIRIP3 as a gene linked to aortic valve stenosis, suggesting its role in cardiac development. While its role in heart conditions is known, its link to AD is less studied (Ignatyeva et al., 2024). This study is the first to associate HIRIP3 with AD, highlighting it as a potential target for developing therapies for AD. The observed differential expression of these genes supports their involvement in the disease process and reinforces the necessity for clinical validation of these biomarkers in human cohorts. Future studies should focus on longitudinal assessments of these biomarkers across different stages of AD to establish their prognostic value and therapeutic implications.

This study underscores the significance of iron metabolism-related gene alterations and immune responses in AD (Peng et al., 2021; Lu et al., 2021). The integration of bioinformatics, machine learning, and experimental validation has provided a multifaceted understanding of the molecular landscape of AD, paving the way for future research aimed at developing effective diagnostic and therapeutic strategies (Zhou et al., 2025). Continued exploration of the interplay between genetic, immune, and metabolic factors will be crucial in addressing the complexities of AD and improving patient outcomes (Bettcher et al., 2021).

The limitations of this study primarily include the lack of mechanistic investigations regarding the key iron metabolism genes identified in AD and the absence of clinical validation analyses. While our bioinformatics approach successfully highlighted potential biomarkers and therapeutic targets, further experimental studies are essential to elucidate the precise roles of these genes in the pathogenesis of AD. Additionally, the reliance on computational models may not fully capture the complexity of biological systems, warranting caution in the interpretation of our findings. Future research should aim to incorporate *in vivo* experiments and clinical data to substantiate the relevance of the identified molecular changes and their implications for AD treatment.

In summary, this research sheds light on the molecular alterations linked to iron metabolism and immune responses in AD, contributing to a deeper understanding of the disease pathology. By identifying key genes and their potential influence on AD progression, our findings lay a foundation for future therapeutic interventions that target iron metabolic and inflammatory pathways. Continued exploration of these biomarkers in clinical contexts will be crucial for translating our findings into effective treatments for Alzheimer’s disease.

## Data Availability

The original contributions presented in the study are included in the article/supplementary material, further inquiries can be directed to the corresponding authors.

## References

[ref1] Bai B. Vanderwall D. Li Y. Wang X. Poudel S. Wang H.. (2021). Proteomic landscape of Alzheimer’s disease: novel insights into pathogenesis and biomarker discovery. Mol. Neurodegener. 16:55. doi: 10.1186/s13024-021-00474-z, PMID: 34384464 PMC8359598

[ref2] Bai B. Wang X. Li Y. Chen P. C. Yu K. Dey K. K.. (2020). Deep multilayer brain proteomics identifies molecular networks in Alzheimer’s disease progression. Neuron 105, 975–991.e7. doi: 10.1016/j.neuron.2019.12.015 31926610 PMC7318843

[ref3] Bettcher B. M. Tansey M. G. Dorothée G. Heneka M. T. (2021). Peripheral and central immune system crosstalk in Alzheimer disease—a research prospectus. Nat. Rev. Neurol. 17, 689–701. doi: 10.1038/s41582-021-00549-x, PMID: 34522039 PMC8439173

[ref4] Boyko M. Stepensky D. Gruenbaum B. F. Gruenbaum S. E. Melamed I. Ohayon S.. (2012). Pharmacokinetics of glutamate-oxaloacetate transaminase and glutamate-pyruvate transaminase and their blood glutamate-lowering activity in naïve rats. Neurochem. Res. 37, 2198–2205. doi: 10.1007/s11064-012-0843-9, PMID: 22846966

[ref5] Celis K. Shuldiner S. Haverfield E. V. Cappell J. Yang R. Gong D.-W.. (2015). Loss of function mutation in glutamic pyruvate transaminase 2 (GPT2) causes developmental encephalopathy. J. Inherit. Metab. Dis. 38, 941–948. doi: 10.1007/s10545-015-9824-x, PMID: 25758935 PMC4919120

[ref6] Chen X. Holtzman D. M. (2022). Emerging roles of innate and adaptive immunity in Alzheimer’s disease. Immunity 55, 2236–2254. doi: 10.1016/j.immuni.2022.10.016, PMID: 36351425 PMC9772134

[ref7] Doki C. Nishida K. Saito S. Shiga M. Ogara H. Kuramoto A.. (2020). Microtubule elongation along actin filaments induced by microtubule-associated protein 4 contributes to the formation of cellular protrusions. J. Biochem. 168, 295–303. doi: 10.1093/jb/mvaa046, PMID: 32289170

[ref8] Feng G. Zhong M. Huang H. Zhao P. Zhang X. Wang T.. (2025). Identification of UBE2N as a biomarker of Alzheimer’s disease by combining WGCNA with machine learning algorithms. Sci. Rep. 15:6479. doi: 10.1038/s41598-025-90578-z, PMID: 39987324 PMC11847011

[ref9] Gao C. Jiang J. Tan Y. Chen S. (2023). Microglia in neurodegenerative diseases: mechanism and potential therapeutic targets. Signal Transduct. Target. Ther. 8:359. doi: 10.1038/s41392-023-01588-0, PMID: 37735487 PMC10514343

[ref10] Giniger E. (2012). Notch signaling and neural connectivity. Curr. Opin. Genet. Dev. 22, 339–346. doi: 10.1016/j.gde.2012.04.003, PMID: 22608692 PMC3426662

[ref11] Graff-Radford J. Yong K. X. X. Apostolova L. G. Bouwman F. H. Carrillo M. Dickerson B. C.. (2021). New insights into atypical Alzheimer’s disease in the era of biomarkers. Lancet Neurol. 20, 222–234. doi: 10.1016/S1474-4422(20)30440-3, PMID: 33609479 PMC8056394

[ref12] Ignatyeva M. Patel A. K. M. Ibrahim A. Albiheyri R. S. Zari A. T. Bahieldin A.. (2024). Identification and characterization of HIRIP3 as a histone H2A chaperone. Cells 13:273. doi: 10.3390/cells13030273, PMID: 38334665 PMC10854748

[ref13] Khan S. Barve K. H. Kumar M. S. (2020). Recent advancements in pathogenesis, diagnostics and treatment of Alzheimer’s disease. Curr. Neuropharmacol. 18, 1106–1125. doi: 10.2174/1570159X18666200528142429, PMID: 32484110 PMC7709159

[ref14] Khemka S. Reddy A. Garcia R. I. Jacobs M. Reddy R. P. Roghani A. K.. (2023). Role of diet and exercise in aging, Alzheimer’s disease, and other chronic diseases. Ageing Res. Rev. 91:102091. doi: 10.1016/j.arr.2023.102091, PMID: 37832608 PMC10842571

[ref15] Koronyo Y. Rentsendorj A. Mirzaei N. Regis G. C. Sheyn J. Shi H.. (2023). Retinal pathological features and proteome signatures of Alzheimer’s disease. Acta Neuropathol. 145, 409–438. doi: 10.1007/s00401-023-02548-2, PMID: 36773106 PMC10020290

[ref16] Kostic M. Zivkovic N. Cvetanovic A. Basic J. Stojanovic I. (2025). Dissecting the immune response of CD4^+^ T cells in Alzheimer’s disease. Rev. Neurosci. 36, 139–168. doi: 10.1515/revneuro-2024-0090, PMID: 39238424

[ref17] Lane D. J. R. Metselaar B. Greenough M. Bush A. I. Ayton S. J. (2021). Ferroptosis and NRF2: an emerging battlefield in the neurodegeneration of Alzheimer’s disease. Essays Biochem. 65, 925–940. doi: 10.1042/EBC20210017, PMID: 34623415

[ref18] Leal M. C. Surace E. I. Holgado M. P. Ferrari C. C. Tarelli R. Pitossi F.. (2012). Notch signaling proteins HES-1 and Hey-1 bind to insulin degrading enzyme (IDE) proximal promoter and repress its transcription and activity: implications for cellular Aβ metabolism. Biochim. Biophys. Acta 1823, 227–235. doi: 10.1016/j.bbamcr.2011.09.014, PMID: 22036964 PMC3307219

[ref19] Leng F. Edison P. (2021). Neuroinflammation and microglial activation in Alzheimer disease: where do we go from here? Nat. Rev. Neurol. 17, 157–172. doi: 10.1038/s41582-020-00435-y, PMID: 33318676

[ref20] Lian P. Cai X. Wang C. Liu K. Yang X. Wu Y.. (2023). Identification of metabolism-related subtypes and feature genes in Alzheimer’s disease. J. Transl. Med. 21:628. doi: 10.1186/s12967-023-04324-y, PMID: 37715200 PMC10504766

[ref21] Liu Y. Tan Y. Zhang Z. Li H. Yi M. Zhang Z.. (2023). Neuroimmune mechanisms underlying Alzheimer’s disease: insights into central and peripheral immune cell crosstalk. Ageing Res. Rev. 84:101831. doi: 10.1016/j.arr.2022.101831, PMID: 36565960

[ref22] Lou J. S. Zhao L. P. Huang Z. H. Chen X. Y. Xu J. T. Tai W. C.. (2021). Ginkgetin derived from *Ginkgo biloba* leaves enhances the therapeutic effect of cisplatin via ferroptosis-mediated disruption of the Nrf2/HO-1 axis in EGFR wild-type non-small-cell lung cancer. Phytomedicine 80:153370. doi: 10.1016/j.phymed.2020.153370, PMID: 33113504

[ref23] Lu Y. Li K. Hu Y. Wang X. (2021). Expression of immune related genes and possible regulatory mechanisms in Alzheimer’s disease. Front. Immunol. 12:768966. doi: 10.3389/fimmu.2021.768966, PMID: 34804058 PMC8602845

[ref24] Ma A. Feng Z. Li Y. Wu Q. Xiong H. Dong M. L.. (2023). Ferroptosis-related signature and immune infiltration characterization in acute lung injury/acute respiratory distress syndrome. Respir. Res. 24:154. doi: 10.1186/s12931-023-02429-y, PMID: 37301835 PMC10257327

[ref25] Machhi J. Yeapuri P. Lu Y. Foster E. Chikhale R. Herskovitz J.. (2021). CD4^+^ effector T cells accelerate Alzheimer’s disease in mice. J. Neuroinflammation 18:272. doi: 10.1186/s12974-021-02308-7, PMID: 34798897 PMC8603581

[ref26] Migliore L. Coppedè F. (2022). Gene-environment interactions in Alzheimer disease: the emerging role of epigenetics. Nat. Rev. Neurol. 18, 643–660. doi: 10.1038/s41582-022-00714-w, PMID: 36180553

[ref27] Ning Z. Liu Y. Guo D. Lin W.-J. Tang Y. (2023). Natural killer cells in the central nervous system. Cell Commun. Signal 21:341. doi: 10.1186/s12964-023-01324-9, PMID: 38031097 PMC10685650

[ref28] Peng Y. Chang X. Lang M. (2021). Iron homeostasis disorder and Alzheimer’s disease. Int. J. Mol. Sci. 22:12442. doi: 10.3390/ijms222212442, PMID: 34830326 PMC8622469

[ref29] Princiotta Cariddi L. Mauri M. Cosentino M. Versino M. Marino F. (2022). Alzheimer’s disease: from immune homeostasis to neuroinflammatory condition. Int. J. Mol. Sci. 23:13008. doi: 10.3390/ijms232113008, PMID: 36361799 PMC9658357

[ref30] Reizis B. (2019). Plasmacytoid dendritic cells: development, regulation, and function. Immunity 50, 37–50. doi: 10.1016/j.immuni.2018.12.027, PMID: 30650380 PMC6342491

[ref31] Rodriguez-Mogeda C. van Ansenwoude C. M. J. van der Molen L. Strijbis E. M. M. Mebius R. E. de Vries H. E. (2024). The role of CD56(bright) NK cells in neurodegenerative disorders. J. Neuroinflammation 21:48. doi: 10.1186/s12974-024-03040-8, PMID: 38350967 PMC10865604

[ref32] Salminen A. Kaarniranta K. Kauppinen A. (2018). The potential importance of myeloid-derived suppressor cells (MDSCs) in the pathogenesis of Alzheimer’s disease. Cell. Mol. Life Sci. 75, 3099–3120. doi: 10.1007/s00018-018-2844-6, PMID: 29779041 PMC11105369

[ref33] Skaper S. D. Facci L. Zusso M. Giusti P. (2017). Neuroinflammation, mast cells, and glia: dangerous liaisons. Neuroscientist 23, 478–498. doi: 10.1177/1073858416687249, PMID: 29283023

[ref34] Song Y. Shi R. Liu Y. Cui F. Han L. Wang C.. (2023). M2 microglia extracellular vesicle miR-124 regulates neural stem cell differentiation in ischemic stroke via AAK1/NOTCH. Stroke 54, 2629–2639. doi: 10.1161/STROKEAHA.122.041611, PMID: 37586072

[ref35] Uddin M. S. Kabir M. T. Jalouli M. Rahman M. A. Jeandet P. Behl T.. (2022). Neuroinflammatory signaling in the pathogenesis of Alzheimer’s disease. Curr. Neuropharmacol. 20, 126–146. doi: 10.2174/1570159X19666210826130210, PMID: 34525932 PMC9199559

[ref36] Um J. H. Shin D. J. Choi S. M. Nathan A. B. P. Kim Y. Y. Lee D. Y.. (2024). Selective induction of Rab9-dependent alternative mitophagy using a synthetic derivative of isoquinoline alleviates mitochondrial dysfunction and cognitive deficits in Alzheimer’s disease models. Theranostics 14, 56–74. doi: 10.7150/thno.88718, PMID: 38164158 PMC10750208

[ref37] Wang J. Fu J. Zhao Y. Liu Q. Yan X. Su J. (2023). Iron and targeted iron therapy in Alzheimer’s disease. Int. J. Mol. Sci. 24:16353. doi: 10.3390/ijms242216353, PMID: 38003544 PMC10671546

[ref38] Wu Y. Hu H. Wang T. Guo W. Zhao S. Wei R. (2024). Characterizing mitochondrial features in osteoarthritis through integrative multi-omics and machine learning analysis. Front. Immunol. 15:1414301. doi: 10.3389/fimmu.2024.1414301, PMID: 39026663 PMC11254675

[ref39] Wu L. Xian X. Tan Z. Dong F. Xu G. Zhang M.. (2023). The role of iron metabolism, lipid metabolism, and redox homeostasis in Alzheimer’s disease: from the perspective of ferroptosis. Mol. Neurobiol. 60, 2832–2850. doi: 10.1007/s12035-023-03245-7, PMID: 36735178

[ref40] Yang K.-F. Zhang J.-Y. Feng M. Yao K. Liu Y.-Y. Zhou M.-S.. (2024). Secretase promotes AD progression: simultaneously cleave notch and APP. Front. Aging Neurosci. 16:1445470. doi: 10.3389/fnagi.2024.1445470, PMID: 39634655 PMC11615878

[ref41] Yang F. Zhang N. Ou G. Y. Xu S. W. (2024). Integrated bioinformatic analysis and validation identifies immune microenvironment-related potential biomarkers in Alzheimer’s disease. J. Prev. Alzheimers Dis. 11, 495–506. doi: 10.14283/jpad.2024.5, PMID: 38374756

[ref42] Zeng J. Liao Z. Yang H. Wang Q. Wu Z. Hua F.. (2024). T cell infiltration mediates neurodegeneration and cognitive decline in Alzheimer’s disease. Neurobiol. Dis. 193:106461. doi: 10.1016/j.nbd.2024.106461, PMID: 38437992

[ref43] Zhang J. Lin F. Xu Y. Sun J. Zhang L. Chen W. (2025). Lactylation and ischemic stroke: research progress and potential relationship. Mol. Neurobiol. 62, 5359–5376. doi: 10.1007/s12035-024-04624-4, PMID: 39541071

[ref44] Zhang Y. Miao Y. Tan J. Chen F. Lei P. Zhang Q. (2023). Identification of mitochondrial related signature associated with immune microenvironment in Alzheimer’s disease. J. Transl. Med. 21:458. doi: 10.1186/s12967-023-04254-9, PMID: 37434203 PMC10334674

[ref45] Zhang P. F. Wang Z. T. Liu Y. Hu H. Sun Y. Hu H. Y.. (2022). Peripheral immune cells and cerebrospinal fluid biomarkers of Alzheimer’s disease pathology in cognitively intact older adults: the CABLE study. J. Alzheimers Dis. 87, 721–730. doi: 10.3233/JAD-220057, PMID: 35342094

[ref46] Zheng Q. Wang X. (2025). Alzheimer’s disease: insights into pathology, molecular mechanisms, and therapy. Protein Cell 16, 83–120. doi: 10.1093/procel/pwae026, PMID: 38733347 PMC11786724

[ref47] Zhou J. Li C. Kim Y. K. Park S. (2025). Bioinformatics and deep learning approach to discover food-derived active ingredients for Alzheimer’s disease therapy. Food Secur. 14:127. doi: 10.3390/foods14010127, PMID: 39796418 PMC11719994

